# High FMNL3 expression promotes nasopharyngeal carcinoma cell metastasis: role in TGF-β1-induced epithelia-to-mesenchymal transition

**DOI:** 10.1038/srep42507

**Published:** 2017-02-15

**Authors:** Yanxia Wu, Zhihua Shen, Keke Wang, Yanping Ha, Hong Lei, Yanan Jia, Ranran Ding, Dongmei Wu, Siyuan Gan, Rujia Li, Botao Luo, Hanguo Jiang, Wei Jie

**Affiliations:** 1 Department of Pathology, Guangdong Medical University, Zhanjiang 524023, China; 2 Department of Pathophysiology, Guangdong Medical University, Zhanjiang 524023, China; 3 Department of Pathology, Union Hospital, Tongji Medical College, Huazhong University of Science and Technology, Wuhan 430030, China

## Abstract

Formin-like 3 (FMNL3) plays a crucial role in cytoskeletal mediation and is potentially a biomarker for cell migration; however, its role in cancer metastasis remains unknown. In this study, we found elevated FMNL3 protein expression in clinical nasopharyngeal carcinoma (NPC) tissues. FMNL3 expression positively correlated to the clinical stage, T (tumour), N (lymph node metastasis) and M (distant metastasis) classification of NPC patients. Moreover, FMNL3 positively correlated to Vimentin expression and negatively correlated to E-cadherin expression in clinical NPC samples. *In vitro* experiments showed that FMNL3 expression was inversely related to NPC cell differentiation status. Overexpression of FMNL3 led to epithelial-to-mesenchymal transition (EMT) in well differentiated CNE1 cells. TGF-β1-treated poorly differentiated CNE2 cells showed changes in EMT accompanied by enhanced FMNL3 expression and cell migration. On the contrary, knockdown of FMNL3 partially attenuated the TGF-β1-promoted CNE2 cell migration, together with associated changes in EMT markers. Finally, knockdown of FMNL3 also weakened EMT in tumours in xenographs. Our study indicates for the first time that TGF-β1/FMNL3 signalling may be a novel mechanism mediating EMT in NPC, which is closely associated with NPC metastasis.

Nasopharyngeal carcinoma (NPC) is a malignant cancer derived from the nasopharyngeal epithelium, with the greatest prevalence in Southern China, especially in Guangdong Province^1^,^2^. There are major three clinical features of NPC: (1) low grade differentiation; the majority of the histological types are non-keratinizing undifferentiated carcinoma with 100% Epstein-Barr virus (EBV) infection; (2) early metastasis; nearly 60% of NPC patients suffer from local lymph node metastasis at first diagnosis; and (3) sensitivity to radiation therapy, but with a high recurrence rate. Conventional radiation therapy has been an effective treatment for NPC. However, local recurrence after radiotherapy is frequent within 2 years post-radiotherapy. Recently, three-dimensional conformal radiation therapy and intensity-modulated radiotherapy have significantly improved the locoregional control of NPC^3^,^4^. However, local recurrence and distant metastasis remain serious complications in the prognosis of NPC patients^5^. Therefore, in addition to better understanding of the metastatic mechanisms of NPC, new NPC metastatic tumour markers should be identified and characterized to assist the rational clinical treatment and prognosis of NPC patients.

Recent studies have shown that epithelial-to-mesenchymal transition (EMT) plays a key role in the invasion and metastasis of various epithelial tumours^6^,^7^. EMT is morphologically characterized by changes from the epithelial cell phenotype into a spindle fibroblast-like appearance and functionally characterized by decreased cell adhesion and increased cell migration. At the molecular level, EMT is associated with a down-regulation of epithelial cell markers (e.g. cytokeratin, E-cadherin, claudins, and occludins) and up-regulation of stromal cell markers (e.g. Vimentin, N-cadherin, matrix metalloproteinases, and fibronectin)^8^,^9^. Among these molecular changes, down-regulation of E-cadherin and up-regulation of Vimentin have been deemed major EMT markers. NPC cells show obvious characteristics of EMT, especially spindle-shaped carcinoma cells^10^. We previously found that inhibition of PI3K/Akt signalling significantly reverses the process of EMT in NPC cells, thereby repressing the pulmonary metastasis of tumour cell-bearing nude nice^11^, which highlights the clinical application of targeting EMT in NPC.

Transforming growth factor-β (TGF-β) is a major regulatory factor of EMT in cancer cells^12^,^13^ that can be secreted by the parenchyma and stromal cells in tumour tissues. The TGF-β cytokine exhibits multiple biological activities that affect cell proliferation, differentiation, apoptosis, and regulation of extracellular matrix production. Increasing evidence has shown that TGF-β has dual functionality in the progression of tumours^14^,^15^. TGF-β may act as a tumour suppressor in the early stages of tumourigenesis, but it functions as a protooncogene at later tumour stages by stimulating angiogenesis and inducing EMT for tumour cell invasion and metastasis. Alterations of serum TGF-β levels and expression of its receptor have been reported in NPC samples^16^,^17^,^18^. However, the underlying molecular mechanism of TGF-β signalling in NPC progression remains to be elucidated.

FMNL3 (formin-like 3, also known as FRL2) is a member of the diaphanous-related formin family, which represents a family of highly conserved cytoskeletal regulatory proteins. Bioinformatics have identified more than 30 members of the formin protein family in plants and 15 members in vertebrates^19^. To date, the limited number of reports has mainly focused on the cloning, evolution, and structural analysis of FMNL3, and little is known about its functions. Harris and colleagues^20^ demonstrated that the FH2 domain of FMNL3 induces generation of filopodia, a cellular structure involved in cell motility. Vega *et al*.^21^ reported that FMNL3 is a downstream effector of RhoC, a member of the Rho family of small G proteins and a effecter of TGF-β signalling^22^, which contributes to actin cytoskeleton reassembly and thus plays an important role in tumour cell invasion and metastasis. Inhibition of FMNL3 expression in prostate carcinoma cells results in a weakened wound-healing ability^23^, and high FMNL3 expression contributes to the progression of colorectal carcinoma^24^. These few reports have supported a possible role of FMNL3 in tumour invasion and metastasis. To date, the role of FMNL3 in NPC remains unknown.

In the present study, we provide evidence that high expression of FMNL3 is associated with the clinical progression of NPC and its EMT status. Furthermore, we found that TGF-β1-induced FMNL3 expression promotes NPC metastasis at least partially via mediating the processes of EMT.

## Results

### FMNL3, E-cadherin and Vimentin expressions and correlation with the clinicopathological features of NPC

**Figure 1:**
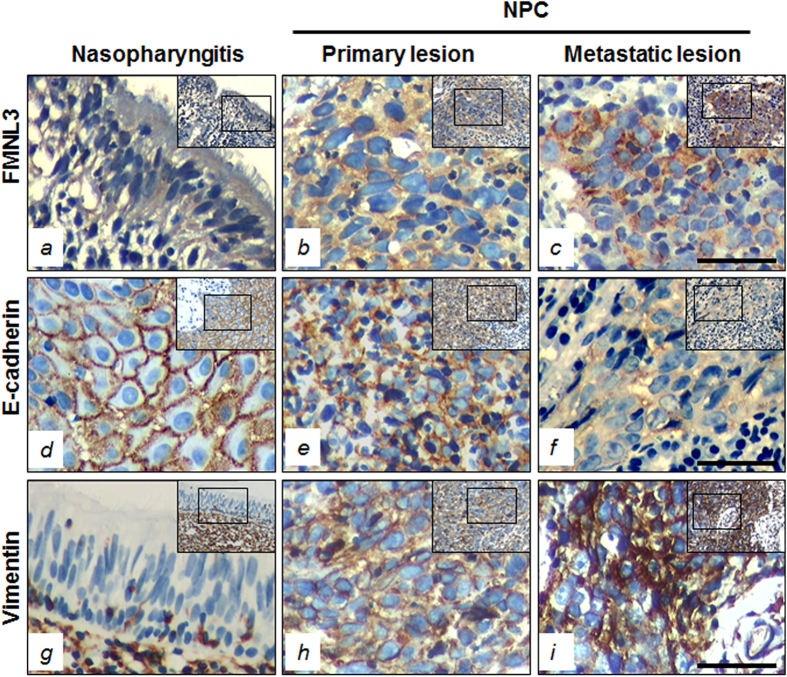
Immunohistochemical staining of FMNL3, E-cadherin, and Vimentin in clinical NPC and NPG samples. FMNL3 expression in NPG cells (**a**), primary NPC cells (**b**) and metastatic NPC cells (**c**). E-cadherin expression in the epithelial cells of NPG samples (**d**), NPC tissues (**e**) and the metastatic samples (**f**). Cytosolic Vimentin protein expression in stromal cells, but not epithelial cells, of nasopharyngitis samples (**g**), and in some tumour cells of primary lesion of NPC (**h**) and the metastatic lesions (**i**). Left panels, NPG; middle panels, NPC primary lesions; right panels, NPC metastatic lesions. Scale bar, 60 μm.

FMNL3, E-cadherin, and Vimentin protein expressions were analysed by immunohistochemistry in tissues from 119 patients with NPC and 29 patients with benign nasopharyngitis (NPG). Positive cytoplasmic expression of FMNL3 protein (+, ++ and +++) was observed in 67.2% (80/119) of NPC patients compared with 6.9% (2/29) of NPG patients (*p* < 0.001) (Fig. 1 and Table 1), suggesting a major role of FMNL3 expression in NPC pathogenesis. E-cadherin, a marker of EMT, showed membrane positivity in 100% (29/29) of epithelial cells in NPG samples, whereas only 36.1% (43/119) of NPC samples were positive for membrane E-cadherin expression (*p* < 0.001) (Fig. 1 and Table 1). Moreover, cytoplasmic positivity for E-cadherin was found in 39.5% (47/119) of NPC patients (Supplementary Figure S1). On the other hand, there was no expression of Vimentin in the epithelial cells of NPG samples (0/29), but stromal cells showed positive expression. However, increased cytosolic Vimentin expression in tumour cells was observed in 68.9% (82/119) of NPC samples (*p* < 0.001) (Fig. 1 and Table 1), especially in the spindle-shaped tumour cells (Supplementary Figure S1).

We next analysed the association of FMNL3, E-cadherin, and Vimentin expression with the clinical parameters of NPC patients. No correlation was observed between FMNL3, E-cadherin, and Vimentin expression with gender, age, or smoking status of NPC patients. However, a positive correlation was found for the clinical classification (I-II vs. III-IV; *p* < 0.001 for FMNL3, membrane E-cadherin, and Vimentin), T (tumour) classification (T1-T2 vs. T3-T4; *p* < 0.01 for FMNL3, *p* < 0.05 for membrane E-cadherin and Vimentin), N (metastasis of lymph node) classification (N0-N1 vs. N2-N3; *p* < 0.05 for FMNL3) and M (distant metastasis) classification (M0 vs. M1; *p* < 0.05 for FMNL3) (Table 1). These results strongly indicate that expression of cytosolic FMNL3 and Vimentin and loss of membrane E-cadherin contributes to the clinical progression of human NPC.

### Association of FMNL3, membrane E-cadherin, and Vimentin in clinical NPC samples

The association of FMNL3, membrane E-cadherin, and Vimentin in clinical NPC samples was further analysed by the Spearman correlation analysis method. We observed a positive correlation of FMNL3 with Vimentin (*r* = 0.236, *p* = 0.01) and a negative correlation of FMNL3 with membrane E-cadherin (*r* = −0.292, *p* = 0.001) and membrane E-cadherin with Vimentin (*r* = −0.207, *p* = 0.024) in NPC samples (Supplementary Tables S1 and S2). These results indicate that FMNL3 expression might be associated with the process of EMT in NPC.

### Expression of FMNL3 and EMT-associated markers in NPC cell lines

**Figure 2:**
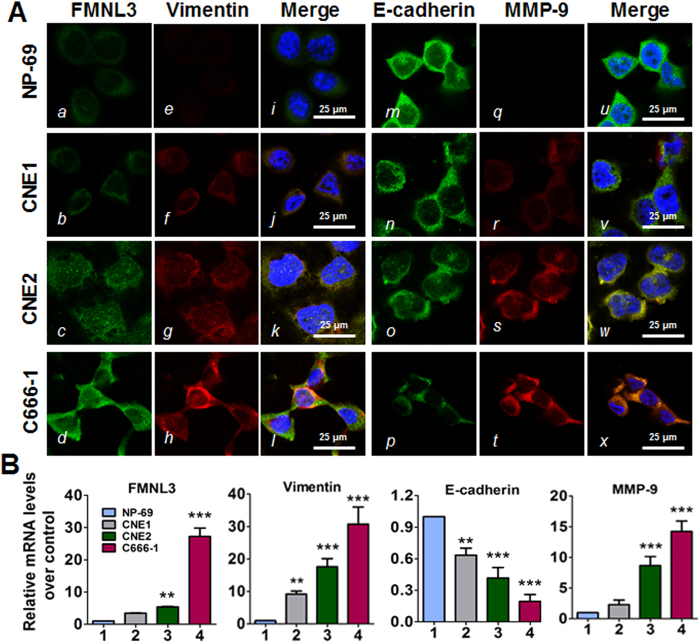
FMNL3 and EMT marker gene expression in various NPC and immortalized NP-69 cells. Well-differentiated CNE1 cells, poorly differentiated CNE2 cells, undifferentiated C666-1 cells, and immortalized non-cancerous NP-69 cells were grown in 6- or 24-well plates (*n* = 3) or on coverslips in 6-cm dishes and then harvested to analyse FMNL3, E-cadherin, Vimentin and MMP-9 expression by immunofluorescence staining (**A**) and qRT-PCR (**B**). For immunofluorescence staining, FMNL3 (a–d), Vimentin (e–h), E-cadherin (m–p) and MMP-9 (q–t) were visualized with FITC- or TRITC-coupled IgGs. The nuclei were counterstained with DAPI. Scale bars, 25 μm. For qRT-PCR, GAPDH was used as a loading control. The levels of the target gene mRNA in NP-69 cells were set as controls. **p < 0.01 vs. NP-69; ***p < 0.001 vs. NP-69.

To further examine the relationship of FMNL3, EMT-associated marker expression with NPC pathogenesis, we analysed three cell lines representing various differentiation stages of NPC. As shown in Fig. 2, the expression of FMNL3, E-cadherin, Vimentin and MMP-9 (matrix metallopeptidase 9) mRNA and protein varied among the NPC cell lines compared with NP-69 immortalized nasopharyngeal epithelial cells. In general, we observed an increase in cytosolic FMNL3, Vimentin and MMP-9 expression and a decrease in membrane E-cadherin expression in well-differentiated CNE1 cells, poorly differentiated CNE2 cells, and undifferentiated C666-1 cells. These results suggest that FMNL3 expression is inversely associated with the cellular differentiation status of NPC and displays a similar trend as Vimentin expression but inverse to membrane E-cadherin expression.

### Overexpression of FMNL3 leads to changes in cellular morphology and EMT markers in CNE1 cells

**Figure 3:**
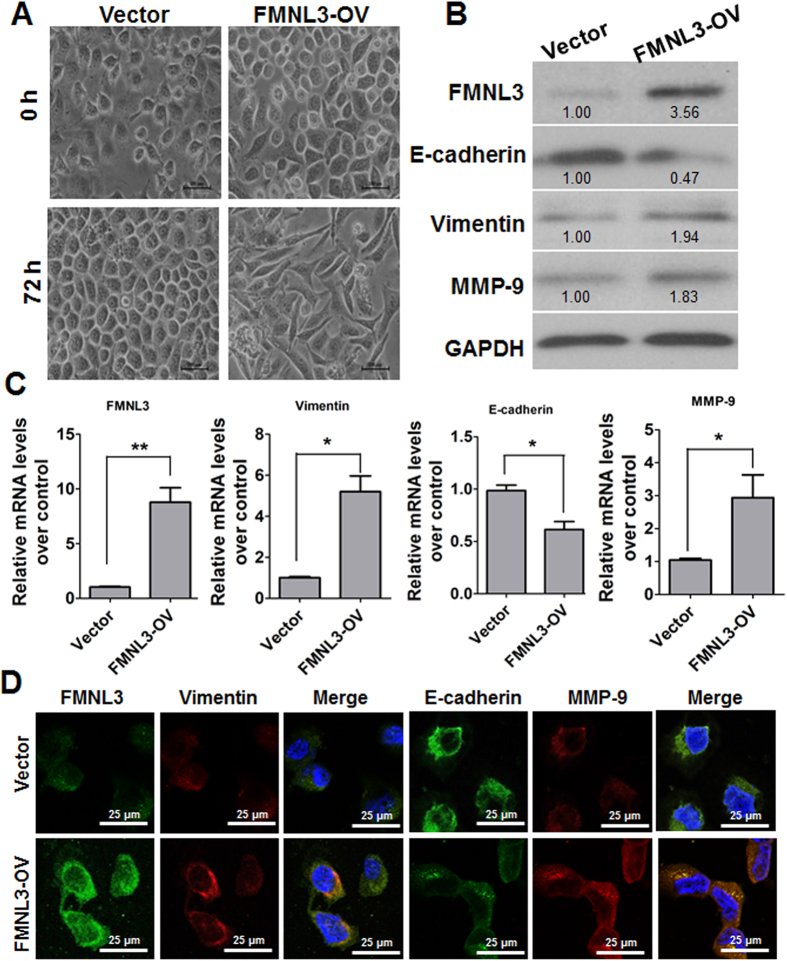
Overexpression of FMNL3 leads to changes in morphology and EMT markers in CNE1 cells. FMNL3-overexpressing vectors (FMNL3-OV) and control vectors were transfected to CNE1 cells. At 72 h later, cell morphology was photographed (**A**) and expressions of EMT marker protein and mRNA were assessed by western blotting (**B**), qRT-PCR (**C**) and immunofluorescent staining (**D**). Scale bars for (**A**), 100 μm, for (**D**), 25 μm. *p < 0.05; **p < 0.01.

We speculated whether FMNL3, as a member of the diaphanous-related formin family, plays a critical role in cancer biology like FMNL2 and FMNL1^25^,^26^. As we expected, overexpression of FMNL3 in well differentiated CNE1 cells resulted in cellular morphological changes that were similar to EMT (Fig. 3A). The molecular changes in the expression of E-cadherin, Vimentin and MMP-9 also supported the occurrence of EMT in FMNL3-overexpressing cells (Fig. 3B–D).

### FMNL3 is involved in TGF-β1-induced EMT in CNE2 cells

**Figure 4:**
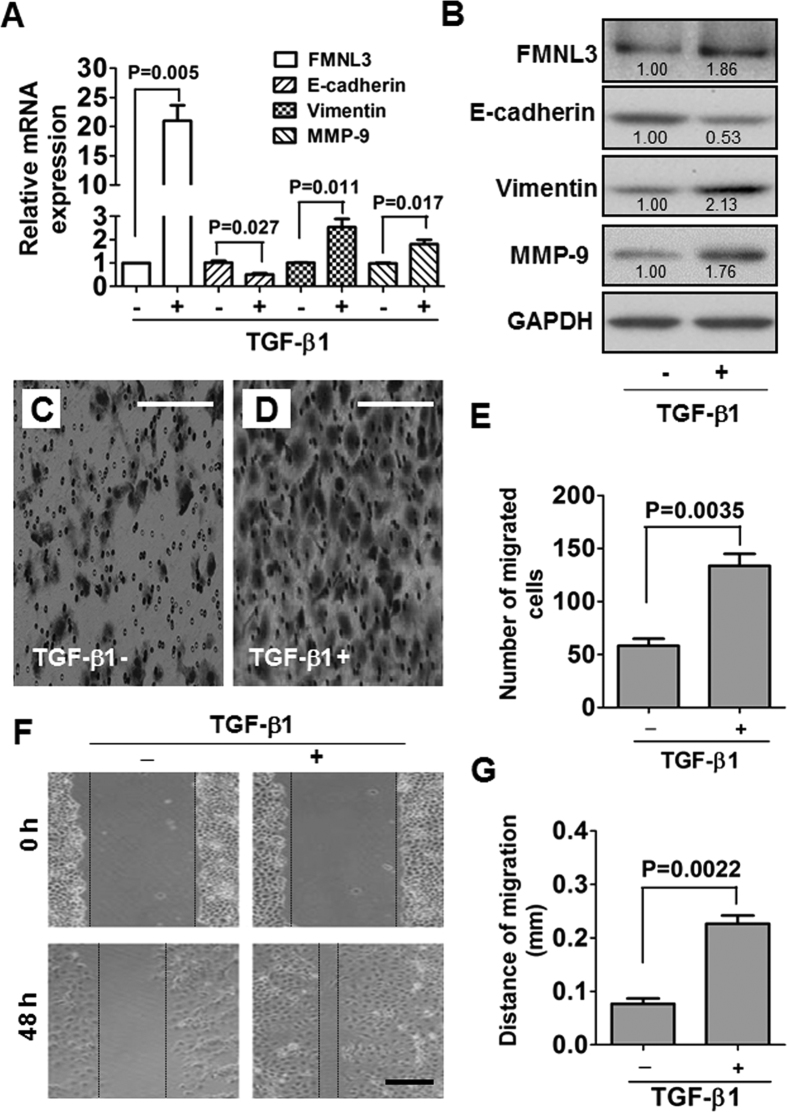
TGF-β1-induced changes in FMNL3 and EMT marker gene expression and cell migration in CNE2 cells. CNE2 cells were treated with 10 ng/ml TGF-β1 for 48 h, and the expressions of FMNL3, Vimentin, E-cadherin and MMP-9 mRNA and protein were examined by qRT-PCR (**A**) and western blotting (**B**). For qRT-PCR and western blotting, GAPDH served as a loading control. After CNE2 cells were treated with 10 ng/ml TGF-β1 for 48 h, cell motility was analysed by a transwell migration assay (**C–E**) and wound healing assay (**F**,**G**). In the transwell migration assay, migrated cells were stained with 0.1% crystal violet. Scale bars, 60 μm (**C**,**D**) and 200 μm (**F**).

TGF-β1 plays a critical role in EMT of many types of cancers^27^,^28^,^29^. Therefore, we examined the morphological and molecular changes in TGF-β1-treated NPC cells. The TGF-β1 receptor 2 (TGFR2) was expressed in all NPC cell lines (Supplementary Figure S3). We thus treated NPC cells with exogenous TGF-β1 and found that TGF-β1 altered E-cadherin and Vimentin expression in CNE2 cells in a time-dependent manner (Supplementary Figure S4). Exogenous TGF-β1 treatment of the poorly differentiated NPC cell line CNE2 led to obvious morphological changes (Supplementary Figure S5). At 48 h post-TGF-β1 treatment, some CNE2 cells began to change into spindle-like cells. After 120 h, part cells changed into obvious mesenchymal-like cells, displaying typical EMT morphology. At the molecular level, we observed up-regulation of FMNL3, Vimentin and MMP-9 and down-regulation of E-cadherin in TGF-β1-treated CNE2 cells (Fig. 4A,B). These results were paralleled by enhancement of cellular migratory ability as measured by transwell migration and wound healing assays (Fig. 4C–G).

### Knockdown of FMNL3 attenuates TGF-β1-facilitated CNE2 cell migration

**Figure 5:**
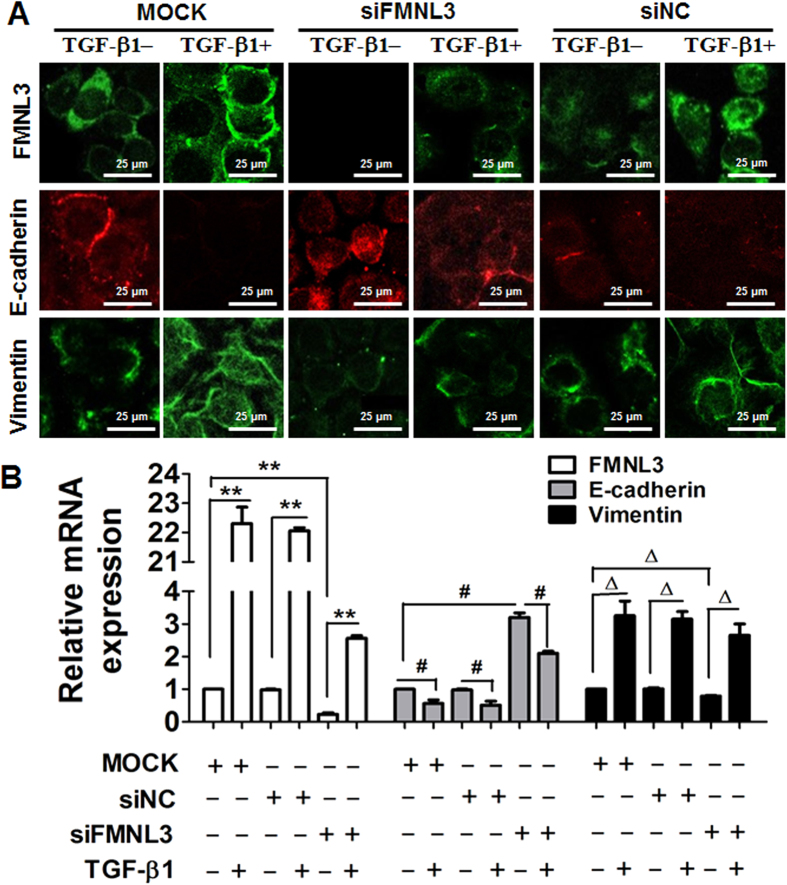
Knockdown of FMNL3 alters FMNL3 and EMT marker gene expression in CNE2 cells. CNE2 cells were transfected with FMNL3-specific siRNA (siFMNL3 oligo 1), control siRNA (siNC), or no siRNA (MOCK) and then treated with or without 10 ng/ml TGF-β1 for 48 h. The cells were harvested and analysed for FMNL3, E-cadherin, and Vimentin mRNA and protein expression by (**A**) Immunofluorescent staining and qRT-PCR (**B**). For immunofluorescent staining, images of E-cadherin and Vimentin photographed from the same field were visualized with FITC- or TRITC-coupled IgGs. For qRT-PCR and western blotting, GAPDH was used as a loading control. ^**^p < 0.01, ^#^*p* < 0.05 and ^Δ^*p* < 0.05.

**Figure 6:**
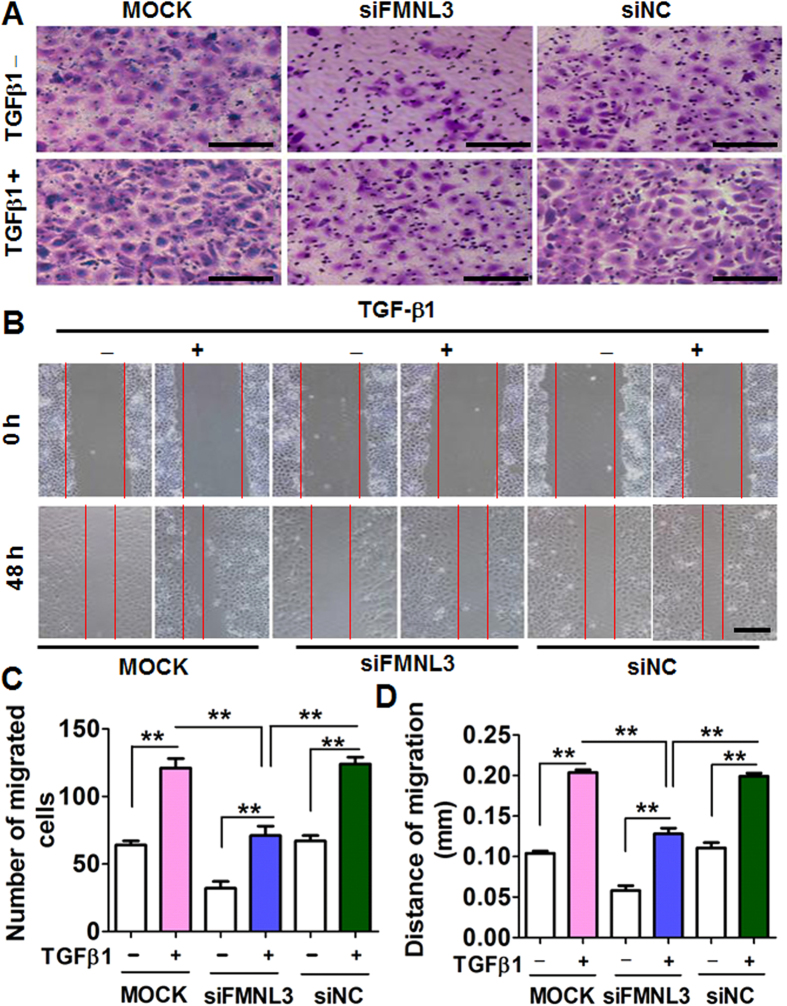
Knockdown of FMNL3 expression suppresses the migratory ability of CNE2 cells. CNE2 cells were transfected with FMNL3-specific siRNA (siFMNL3 oligo 1), control siRNA (siNC), or no siRNA (MOCK) and then treated with or without TGF-β1 (10 ng/ml) for 48 h. The cells were harvested and used in transwell migration assays (**A**,**C**) and wound healing assays (**B**,**D**). Untreated CNE2 cells served as controls. Each group was analysed in three wells (*n* = 3). Scale bars, 60 μm (**A**) and 200 μm (**B**). **p < 0.01.

To assess the role of FMNL3 in NPC cell migration, we used three pair of small interfering (si) RNAs (oligo 1, 2 and 3) specific for FMNL3 to knockdown FMNL3 expression in CNE2 cells. The initial results showed all three well designed oligoes attenuated FMNL3 mRNA significantly, and we chose oligo 1 that with the most effective inhibition to be used in the associated experiments; besides, the specificity of the oligo 1 was rigorously assessed (Supplementary Figure S6). At mRNA level, we found that knockdown of FMNL3 led to increase of E-cadherin and decrease of Vimentin in CNE2 cells without addition of TGF-β1 when compared with controls, which were reversed by a certain extent after TGF-β1 addition (Fig. 5B), and these trends in changes were further indicated by protein levels (Fig. 5A and Supplementary Figure S7). In parallel with changes in expressions of FMNL3, E-cadherin and Vimentin, knockdown of FMNL3 expression in CNE2 cells also led to attenuation of cell migration ability. While TGF-β1 stimulation enhanced cell migration of CNE2 cells transfected with control siRNA, we observed partial suppression of cell migration in cells transfected with FMNL3-specific siRNA, to some extent (Fig. 6). To avoid off-target effects by single siRNA, oligo 2 was additionally used in migration assays, and results showed that oligo 2 specific for FMNL3 mRNA also led to attenuated cell migration (Figure S8). Interestingly, the change of cell function was consistent with the trends of cytoskeletal F-actin distribution in NPC cells (Figure S9). These results supported a functional role of FMNL3 in NPC cell migration.

### Knockdown of FMNL3 weakens EMT in tumour cells in xenographs

**Figure 7:**
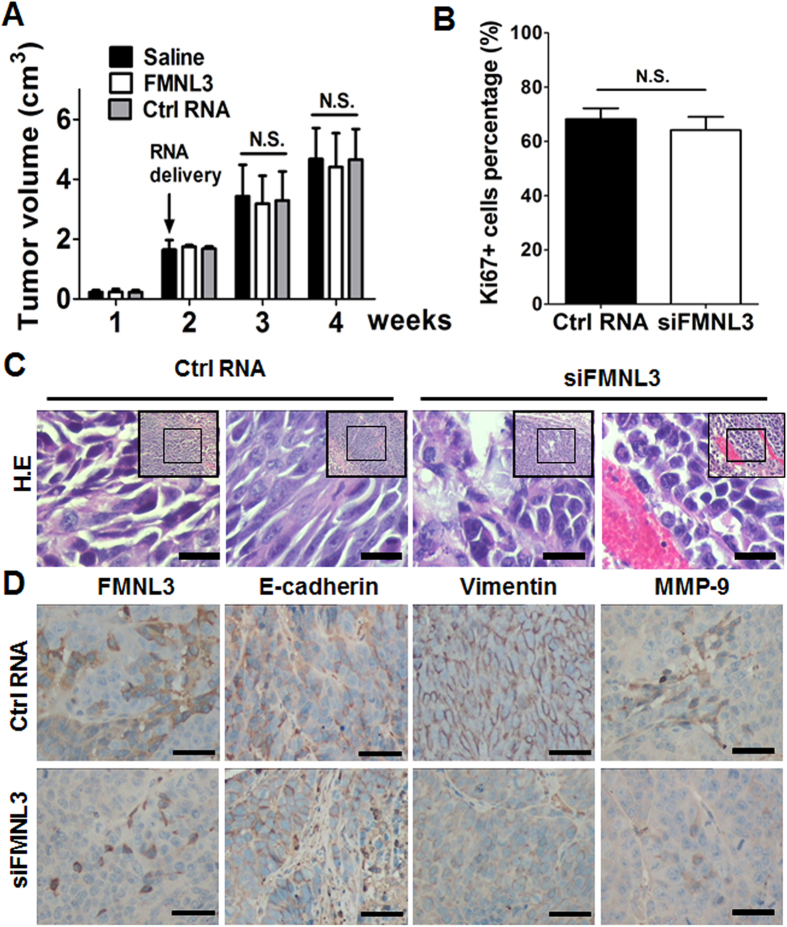
Knockdown of FMNL3 suppresses EMT *in vivo*. CNE2 cells were subcutaneously injected into the flank of nude mice and xenographs were formed 2 weeks later. Cholesterol-conjugated FMNL3-specific siRNAs (FMNL3 oligo 1) or -control siRNAs (ctrl RNA) were intratumorally injected; saline was used as a control. At the end of time points, mice were euthanized and xenographs were obtained for further analysis. (**A**) Tumour volumes. (**B**) Percentages of Ki67 + cells detected by immunohistochemical staining were used to assess proliferation of tumour cells in xenographs. (**C**) Representative photographs of cell morphology assessed by hematoxylin and eosin (H.E) staining. (**D**) FMNL3 and EMT markers (E-cadherin, Vimentin and MMP-9) were detected by immunohistochemical staining. Scale bars, 30 μm (**C**) and 60 μm (**D**).

CNE2 cells were subcutaneously injected into nude mice and two weeks later at formation of xenographs, cholesterol-conjugated siRNAs were intratumourally injected to suppress FMNL3 expression. Inhibition of FMNL3 *in vivo* showed no significant effects on tumour proliferation, as evidenced by tumour volume and Ki67 index measurements (Fig. 7A and B). Microscopically, some tumour cells in xenographs subjected to control RNA treatment displayed mesenchyme-like morphology, while tumour cells with siFMNL3 treatment displayed epithelioid morphology (Fig. 7C). Interestingly, mesenchyme-like tumour cells showed strong FMNL3 expression while epithelioid tumour cells exhibited slight FMNL3 expression. Consistent with the *in vitro* experiments results, knockdown of FMNL3 *in vivo* also led to up-regulation of E-cadherin and down-regulation of Vimentin and MMP-9 in tumour cells in xenographs (Fig. 7D). Grossly and microscopically, we did not observe any metastatic lesions in lung and liver in all mice (data not shown).

## Discussion

NPC is the most common malignant carcinoma derived from the nasopharyngeal mucosa with regional distribution characteristics^1^. In the present study, we delineated the role of FMNL3, a mediator of actin cytoskeleton formation^26^, in the clinical progression of NPC. We found that FMNL3 expression was much higher in NPC than that in NPG (Fig. 1 and Table 1). In addition, positive FMNL3 expression was associated with the clinical, T, N and M classifications of NPC samples (Table 1). These preliminary results indicate that high FMNL3 expression contributes to the clinical progression of NPC.

EMT is one of the key factors involved in tumour progression^30^,^31^,^32^. NPC cells display characteristics of EMT^10^,^33^,^34^, particularly the neoplastic spindle-shaped cells^10^. Recently, we reported that inhibition of PI3K/Akt signalling represses EMT, thus attenuating pulmonary metastasis of NPC cells^11^, which highlights the possibility of EMT blockade for the treatment of NPC metastasis. Much recently, Gauvin and colleagues reported that FMNL3 and N-cadherin co-localized in leading edges and cell-cell contact sites of 3T3 cells^35^. N-cadherin, like Vimentin, is a mesenchymal marker, and the increase of N-cadherin and decrease of membrane E-cadherin is a common molecular event during EMT in cancer. In the present study, we found that NPCs underwent EMT, as indicated by overexpression of Vimentin and down-regulation of membrane E-cadherin in clinical NPC samples (Fig. 1 and Table 1). Furthermore, this expression pattern was associated with the clinical and T classifications of NPC (Table 1). Spearman correlation analysis showed that FMNL3 expression was positively correlated to Vimentin expression but negatively correlated to membrane E-cadherin expression in clinical NPC samples (Supplementary Table S1). Taken together, these results suggested that high expression of FMNL3 was closely associated with EMT in NPC samples, indicating that FMNL3 participates in the progression of NPC.

We next screened FMNL3 expression in NPC cell lines with various differentiation statuses. NP-69 immortalized nasopharyngeal epithelial cells displayed basal FMNL3 expression, whereas CNE1, CNE2 and C666-1 cells showed increases in FMNL3 expression (Fig. 2). In general, FMNL3 expression level was inversely associated with NPC cell differentiation status. In cells inhibited for FMNL3 expression, we observed a significant abrogation of cell migration (Fig. 6). These results were consistent with the expression profiles of FMNL3 in clinical NPC samples. In line with the differentiation status of NPC cell lines, down-regulation of membrane E-cadherin and up-regulation of Vimentin and MMP-9 indicated that these NPC cell lines had undergone EMT, especially the fibroblast-like C666-1 cells. Considering well differentiated CNE1 cells displayed a low level of FMNL3 expression, we thus forced FMNL3 expression in CNE1 cells, and we observed a typical morphology of EMT in FMNL3-overexpressing cells, this morphological changes were paralleled with associated EMT markers expression (Fig. 3), indicating the sufficiency of FMNL3 in inducing EMT in NPC cells.

Because TGF-β1 signalling promotes EMT in many types of carcinomas, we thus examined TGF-β1-treated NPC cells. TGF-β1 treatment of poorly-differentiated CNE2 cells resulted in typical morphological changes of EMT in a time-dependent manner (Supplementary Figure S5), and these changes were accompanied by enhanced cell migration (Fig. 4C–G). At the molecular level, the enhanced FMNL3 and Vimentin expression and decreased E-cadherin expression were consistent with the morphological changes of TGF-β1-treated CNE2 cells (Fig. 4A,B, Supplementary Figure S4). Activation of TGF-β1 signalling can influence cell proliferation, differentiation, and apoptosis in cell type- and context-dependent manners^36^,^37^,^38^. In the present study, we did not find any proliferation-promoting effects of TGF-β1 on NPC cells (Supplementary Figure S10). These results demonstrated that TGF-β1 sufficiently induced EMT in NPC cells. Furthermore, siRNA-mediated inhibition of FMNL3 attenuated TGF-β1-induced cell migration compared with TGF-β1-treated cells transfected with control siRNA (Fig. 6). These functional changes were consistent with the changes of FMNL3 with EMT markers *in vitro* (Fig. 5 and Figure S7), *in vivo* (Fig. 7), and with regards to the F-actin distribution (Figure S9).

Progression to metastatic disease is generally accompanied by altered TGF-β responsiveness and increased expression or activation of TGF-β^15^. To examine the activity of the TGF-β1 pathway in NPC samples, we determined the serum TGF-β1 levels in clinical NPC patients using ELISA. We found higher serum TGF-β1 levels in pre-radiotherapeutic NPC patients compared with healthy controls, and the levels were decreased post radiotherapy (Figure S2), implying a positive role of TGF-β1 in the clinical progression in NPC patients. We further examined TGF-β1 and one of its receptors TGFR2 in NPC tissues *ex vivo* and found a down-regulation of TGF-β1 in tumour cells and up-regulation of TGFR2 in clinical NPC samples. TGFR2 was also presented on the NP-69, CNE1, CNE2 and C666-1 cell lines (Supplementary Figure S3). This observation suggests an alteration in TGF-β1 signalling in the progression of NPC. Changes in serum TGF-β1 may also reflect the immunological switch in cancer patients^39^. Moreover, a previous report showed that EBV infection of cancer patients might influence serum TGF-β1 levels^40^,^41^. We did not assess the EBV infection status of patients involved in our study, although we will include this parameter in future studies.

FMNL3 is closely associated with actin polymerization^42^,^43^, and therefore regulates cell migration. Recently, Zeng *et al*. reported that high FMNL3 expression mediates progression and metastasis in colorectal carcinoma^23^; however, its mechanism remains unknown. As a member of the diaphanous-related formin subfamily, FMNL2 has been proposed to be involved in cancer metastasis through TGF-β1/Smad signalling^25^. Because our current investigation found a close relationship between TGF-β1 stimuli and FMNL3 expression, we hypothesized that TGF-β1/Smad signalling may be involved in FMNL3-mediated EMT in NPC cells. This hypothesis should be examined in future studies. Considering the high serum TGF-β1 levels in NPC patients, we hypothesize that under such microenvironments, NPC cells gradually increase FMNL3 expression and then undergo remodelling of cellular cytoskeleton and adhesion, and eventually the cancer cells acquire high metastatic potential as a result of EMT.

There are some limitations of our present investigation. We provided *in vitro* evidence to support that TGF-β1/FMNL3 signalling contributes to the EMT of NPC cells. However, the animal experiments did not show signs of metastasis; this may be a result of the short treatment periods. Considering that TGF-β1 signalling cross-talks with other signalling pathways such as RhoC^44^, we should further determine the mechanism of TGF-β1-induced FMNL3 expression in NPC. In addition, follow-up data on the NPC patients would strengthen the significance of our findings.

## Conclusions

Our study provides the first evidence that TGF-β1/FMNL3 signalling contributes to NPC metastasis via mediating EMT processes, which may be a new mechanism of NPC clinical progression.

## Methods

### Ethics statement

The use of human tissue samples in this study was approved by the Ethics Council of the Affiliated Hospital of the Guangdong Medical University (Zhanjiang, China) for Approval of Research Involving Human Subjects. Ethical guidelines under the Declaration of Helsinki were followed. Written informed consent was obtained from all subjects for publication of this study. All experiments were performed in accordance with approved guidelines of Guangdong Medical University.

### Patients and specimens

Paraffin-embedded samples were obtained from 148 patients at the Affiliated Hospital of Guangdong Medical University during 2008–2013. Patients had not received preoperative radiotherapy or chemotherapy. The 148 patients included 119 NPC cases (81 men and 38 women) and 29 NPG cases (18 men and 11 women). Clinical data of the NPC patients were reviewed based on the pathological tumour-node-metastasis system (AJCC/UICC 2002). NPC patients were first diagnosed at a median age of 48 years (range, 18–75 years). All NPC patients were diagnosed with non-keratinizing carcinoma following histological examination.

### Immunohistochemical staining

Immunohistochemistry was performed to test target proteins expression in samples from clinical patients and tumour xenographs by standard protocols. Briefly, paraffin sections (4 μm) were deparaffinized and rehydrated, and heat-induced antigen retrieval was conducted in sodium citrate buffer (10 mM, pH 6.0). Endogenous peroxidases were blocked by incubation in 0.3% H_2_O_2_. The sections were then incubated with primary antibodies against FMNL3 (Cat. #HPA023201; Sigma, Germany), E-cadherin (Cat. #3195 S; Cell Signaling, MA, USA), Vimentin (Cat. #5741 S; Cell Signaling) and MMP-9 (Cat. #sc-21733; Santa Cruz) at 4 °C overnight. Non-immune IgG was used as a negative control. Antigenic sites were visualized using a SP9000 and DAB kits (ZSGB-BIO, Beijing, China). The immunoreactive scores (IRS) of FMNL3, E-cadherin, Vimentin and MMP-9 were calculated as follows: 0, negative; 1, weak; 2, moderate; or 3, strong. The percentage of positive cells was scored as follows: 1, 0–9% positive cells; 2, 10–50% positive cells; and 3, >50% positive cells. The two scores were multiplied together and samples with a total IRS of 0, 1–3, 4–6 and 7–9 were considered as (−), (+), (++) and (+++), respectively.

### Cell culture

NPC cell lines CNE1 (well differentiated, EBV−), CNE2 (poorly differentiated, EBV−), and C666-1 (undifferentiated, EBV +) and the immortalized nasopharyngeal epithelial cell line NP-69 (EBV−) were maintained as described previously^11^,^45^,^46^. Briefly, all NPC cell lines were cultured in Dulbecco’s modified Eagle’s medium (DMEM; HyClone) supplemented with 10% heat-inactivated fetal bovine serum (FBS; HyClone), 100 U/ml penicillin, and 100 μg/ml streptomycin. NP-69 cells were cultured in defined keratinocyte serum-free medium (Cat. #10744-019, Life Technologies, CA, USA) supplemented with 5% heat-inactivated FBS, 100 U/ml penicillin, 100 μg/ml streptomycin, and 0.2 ng/ml recombinant epidermal growth factor. Cells were cultured at 37 °C in a humidified atmosphere with 5% CO_2_. Medium was changed every 2 days.

### FMNL3 knockdown and overexpression

For FMNL3 knockdown experiments, we used three pairs of specific siRNA targeting human FMNL3 [NCBI: NM_175736.4] purchased from RiboBio Co., Ltd. (Guangzhou, China). The siRNA sequences were as follows: oligo 1, 5′-CUGUCAGCCAUUCGAAUUAdTdT-3′, 5′-UAAUUCGAAUGGCUGACAGdTdT-3′; oligo 2,5′-GCACUUAGCCUCAAUAACAdTdT-3′, 5′-UGUUAUUGAGGCUAAGUGCdTdT-3′; oligo 3,5′-GUAGUAUGAGUUUACCAAGdTdT-3′, 5′-CUUGGUAAACUCAUACUGCdTdT-3′. The specificity of the FMNL3 siRNA was rigorously analysed by bioinformatics, especially for absence of binding to FMNL2 (NM_052905.3) and FMNL1 (NM_005892.3). A scrambled sequence (si-Control; Cat. #siB05815) was used as a negative control. For FMNL3 overexpression experiments, a FMNL3-overexpressing vector (Cat.#EX-Y4459-M98-5, CMV-T7-ORF-eGFP-Neo) and negative control vector (Cat.#EX-NEG-M98, CMV-T7-eGFP-Neo) were purchased from GeneCopoeia (Rockville, MD, USA). One day prior to transfection, CNE1 and CNE2 cells were seeded in 6- or 24-well plates in complete medium. Subconfluent (50–60%) cells were transfected with siRNAs or vectors using Lipofectamine™ 2000 (Life Technologies, NY, USA) in Opti-MEM (Life Technologies). Following incubation of the cells at 37 °C in a humidified atmosphere with 5% CO_2_ for 6 h, the medium was replaced with complete cell culture medium. Transfection efficiency was confirmed by fluorescence microscopy of cells transfected with Cy3-conjugated control oligos or eGFP-tagged control vectors.

### Transwell migration and wound healing assays

For the transwell migration assay, we used Transwell chambers with 8-μm polycarbonate membranes (BD Biosciences, NJ, USA). After transfection of CNE2 cells with si-FMNL3, si-Control, or no siRNA for 24 h, cells were treated with or without 10 ng/ml TGF-β1 (Proptech) for 48 h. Cells were harvested, resuspended in DMEM supplemented with 0.5% FBS and seeded in the upper chamber (1 × 10^5^ cells/well). DMEM supplemented with 10% FBS was added to the lower chamber. Migration was allowed to proceed for 24 h at 37 °C, and then the membranes were fixed for 20 min with 4% neutral formalin and stained with 0.1% crystal violet for 5 min. The membranes were washed with PBS and cut from the inserts. Cells on the upper surface of the membrane were removed with a cotton swab, and the membranes were mounted with glycerol. The number of migrated cells on the lower surfaces of the membranes was determined by counting 15 representative fields (20 × objective) in triplicate inserts for each group.

For the wound healing assay, CNE2 cells (5 × 10^4^) transfected with si-FMNL3, si-Control, or no siRNA for 24 h were harvested and seeded onto 6-well plates in DMEM with 0.5% FBS and cultured for 24 h. When the cells reached 80% confluence, three parallel scratches were made using a 10-μl pipette tip. The cells were washed with 1× PBS and treated with or without 10 ng/ml TGF-β1 for an additional 48 h. Images were captured at 0 and 48 h post-scratching. Image J software was used to detect the migrated distances of the cells. Triplicate wells were used for each treatment.

### RNA extraction and qRT-PCR

Total RNA was extracted with Trizol reagent (Life Technologies). cDNA was synthesized from 1 μg total RNA using an oligo(dT) 18 primer and a PrimeScript® RT reagent Kit (TaKaRa, Dalian, China). qRT-PCR was performed using the following primer pairs (5′–3′): FMNL3 (NM_175736.4), forward, CAGCGAACTTGATGATGAGAAG, FMNL3 reverse, TCTTGTTTTTGGAGCAGA TGAG; E-cadherin (NM_004360.3) forward, TTGCTACTGGAACAGGGACAC, E-cadherin reverse, CCCGTGTGTTAGTTCTGCTGT; Vimentin (NM_003380.3) forward, TGCGTGAAATGGAAGAGAACT, Vimentin reverse, TCAGGTTTCAGGGAGGAAAAGT; MMP-9 (NM_004994.2) forward, GGACAAGCTCTTCGGCTTCT, MMP-9 reverse, TCGCTGGTACAGGTCGAGTA; and GAPDH (NM_002046) forward, GTCAACGGATTTGGTCGT, GAPDH reverse, TTGATTTTGGAGGGAT CTCG. PCR was conducted using a LightCycler480 instrument (Roche) in a final volume of 20 μl, including 10 μl SYBR Green I PCR Master Mix (TOYOBO, Osaka, Japan), 0.4 μl forward primer (10 μM), 0.4 μl reverse primer (10 μM), 2 μl cDNA, and 7.2 μl dH_2_O. PCR amplification was performed as follows: 95 °C for 1 min and then 45 cycles of 95 °C for 5 s and 60 °C for 20 s. The relative abundance of target mRNAs was determined from the CT values and plotted as the fold change compared with the control groups. GAPDH served as the loading control.

### Western blotting

Cells were collected and lysed with RIPA lysis buffer. Total proteins (30–50 μg) were subjected to SDS-polyacrylamide electrophoresis and then transferred to polyvinylidene difluoride membranes. After two washes with TBST, the membranes were incubated with 5% skim milk powder in TBST at 37 °C for 1 h and then primary antibodies (FMNL3: 1/1000, Cat. #NBP2-24724, NovusBio, USA; E-cadherin: 1/1000, Cell Signaling; Vimentin, 1/1000, Cell Signaling; MMP-9, 1/1000, Cell Signaling; GAPDH, 1/1000, Cell Signaling) at 4 °C overnight. After two washes with TBST, the membranes were incubated with horseradish peroxidase-conjugated secondary antibodies for 1 h at 37 °C. Bands were visualized using enhanced chemiluminescence reagents (Thermo Fisher, Rockford, IL, USA) and analysed with a gel analysis system (VersDoc TM5000MP System; BIO-RAD, Guangzhou, China). The expression of GAPDH was used as a loading control.

### Indirect immunofluorescence assay

Indirect immunofluorescence was performed using cells grown on glass coverslips as described previously^45^. The cells were incubated overnight at 4 °C with primary antibodies against FMNL3 (Santa Cruz Biotechnology, USA), E-cadherin (Cell Signaling), Vimentin (Cell Signaling) and MMP-9 (Santa Cruz). After two washes with 1 × PBS, antigenic sites were visualized using FITC- or TRITC-conjugated donkey anti-goat or –rabbit IgG (Protein Tech, USA). Images were captured using a laser scanning confocal microscope (TCS SP5; Leica, Germany).

### Tumour xenograph experiments

Tumour xenograph experiments were performed as described previously^11^,^47^. Specific pathogen-free (SPF) Balb/c null mice (8 weeks old) were purchased from the Guangdong Medical Laboratory Animal Center (Foshan, China). All animal procedures were conducted in accordance with protocols approved by the Institutional Animal Care and Use Committee (IACUC) of Guangdong Medical University. CNE2 cells (2 × 10^6^ cells in 200 μl/mouse) were subcutaneously injected into the right flanks of mice. After 2 weeks, tumour-bearing mice were intratumourally injected twice a week with saline (*n* = 5), cholesterol-conjugated siFMNL3 (RiboBio) (4 nmol/20 g; *n* = 6) or cholesterol-conjugated control siRNA (siNC) (RiboBio) (4 nmol/20 g; *n* = 5) for 3 weeks. Mice were monitored, with body weight and tumour sizes measured twice a week. All mice were euthanized at the end of the treatment period. The metastatic lesions in lung and liver were observed. Xenographs were fixed in natural formalin, and embedded in paraffin for immunohistochemical analysis of FMNL3 and EMT markers. The tumour volumes were calculated as (A × B^2^)/2, in which A is the largest diameter, and B is the shortest diameter.

### Statistical analyses

Statistical analyses were performed using SPSS 17.0 (SPSS Inc., Chicago, IL, USA). χ2 analysis was used to analyse comparisons of FMNL3, E-cadherin, and Vimentin expression with the clinical parameters of clinical samples. Spearman correlation analysis was used to analyse correlations of the expression of FMNL3 with E-cadherin and Vimentin in NPC samples. Data from *in vitro* experiments were expressed as the mean ± SD. To analyse differences between multiple groups, analysis of variance was performed with a post-hoc least square difference test. *P-*values of <0.05 were considered statistically significant.

## Additional Information

**How to cite this article:** Wu, Y. *et al*. High FMNL3 expression promotes nasopharyngeal carcinoma cell metastasis: role in TGF-β1-induced epithelia-to-mesenchymal transition. *Sci. Rep.* **7**, 42507; doi: 10.1038/srep42507 (2017).

**Publisher's note:** Springer Nature remains neutral with regard to jurisdictional claims in published maps and institutional affiliations.

## Supplementary Material

Supplementary Information

## Figures and Tables

**Table 1:** Expressions of FMNL3, E-Cadherin and Vimentin in clinical samples and associations with parameters.

Items	FMNL3			E-cadherin			Vimentin		
	−	+ ~ +++	*P* value	−	+ ~ +++	*P* value	−	+ ~ +++	*P* value
Histological type									
NPC	39	80	0.000*	76	43	0.000*	37	82	0.000*
NPG	27	2		0	29		29	0	
Age									
<50	21	45	0.804	42	24	0.954	21	45	0.849
≥50	18	35		34	19		16	37	
Gender									
Male	24	57	0.286	51	30	0.765	26	55	0.729
Female	15	23		25	13		11	27	
Smoking									
Yes	18	47	0.195	42	23	0.852	22	43	0.476
No	21	33		34	20		15	39	
Clinical classification									
I-II	13	4	0.000*	4	13	0.000*	11	6	0.001*
III-IV	26	76		72	30		26	76	
T classification									
T1-T2	23	26	0.006*	25	24	0.015*	21	28	0.020*
T3-T4	16	54		51	19		16	54	
N classification									
N0-N1	19	23	0.032*	23	19	0.127	16	26	0.223
N2-N3	20	57		53	24		21	56	
M classification									
M0	35	57	0.024*	61	31	0.307	27	65	0.448
M1	4	23		15	12		10	17	

NPG, nasopharyngitis; *significance as indicated.
