# Author Correction: High FMNL3 expression promotes nasopharyngeal carcinoma cell metastasis: role in TGF-β1-induced epithelia-to-mesenchymal transition

**DOI:** 10.1038/s41598-022-09013-2

**Published:** 2022-03-25

**Authors:** Yanxia Wu, Zhihua Shen, Keke Wang, Yanping Ha, Hong Lei, Yanan Jia, Ranran Ding, Dongmei Wu, Siyuan Gan, Rujia Li, Botao Luo, Hanguo Jiang, Wei Jie

**Affiliations:** 1 Department of Pathology, Guangdong Medical University, Zhanjiang, 524023 China; 2 Department of Pathophysiology, Guangdong Medical University, Zhanjiang, 524023 China; 3 Department of Pathology, Union Hospital, Tongji Medical College, Huazhong University of Science and Technology, Wuhan, 430030 China

Correction to: *Scientific Reports* 10.1038/srep42507, published online 15 February 2017

This Article contains an error in Figure 6B, where the incorrect image was used for siNC (TGFβ1-, 48 h). The correct Figure 6 and its accompanying legend appear below.

**Figure 6:**
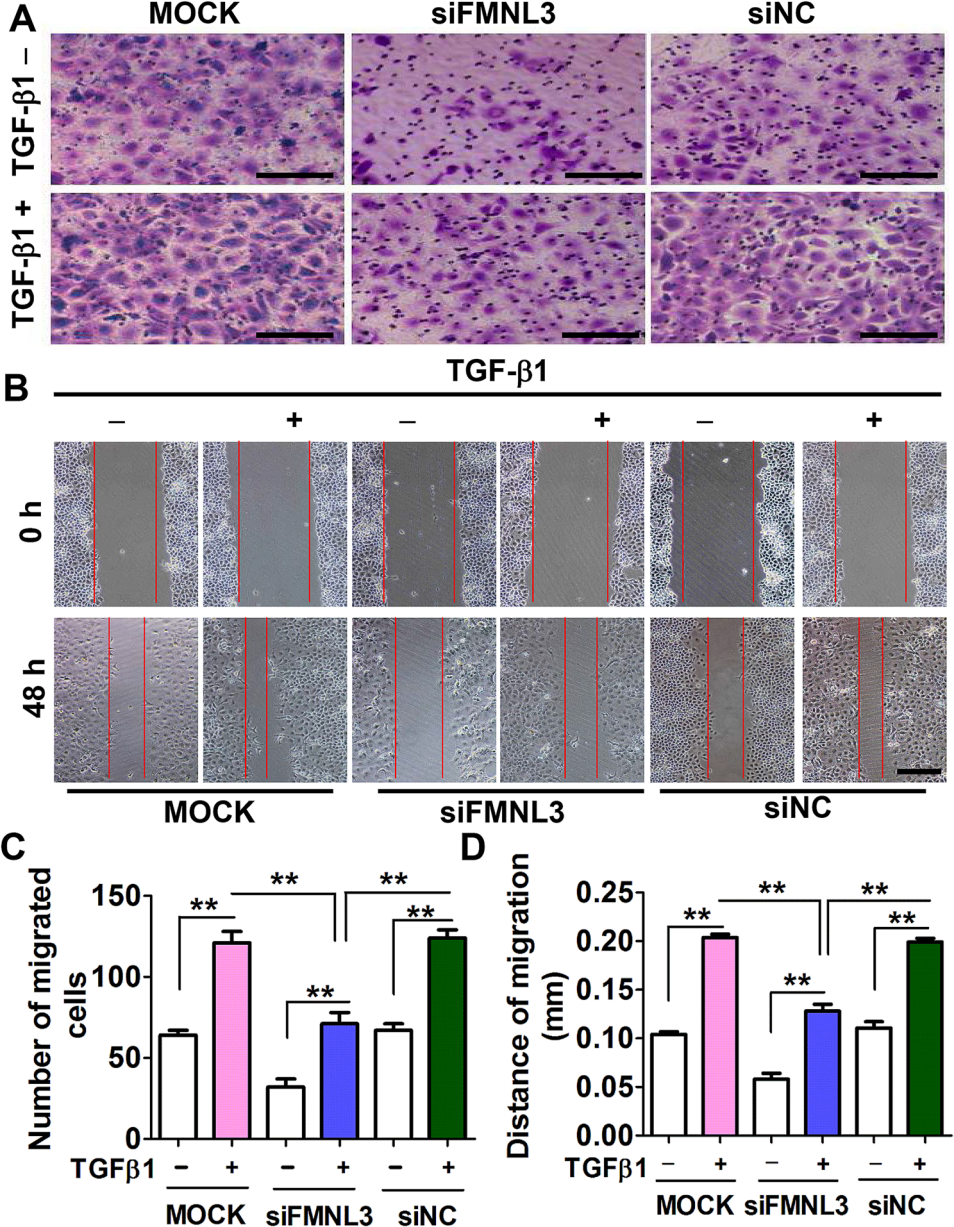
CNE2 cells were transfected with FMNL3-specific siRNA (siFMNL3 oligo 1), control siRNA (siNC), or no siRNA (MOCK) and then treated with or without TGF-β1 (10 ng/ml) for 48 h. The cells were harvested and used in transwell migration assays (**A**,**C**) and wound healing assays (**B**,**D**). Untreated CNE2 cells served as controls. Each group was analysed in three wells (*n* = 3). Scale bars, 60 μm (**A**) and 200 μm (**B**). **p < 0.01.

These changes do not affect the conclusions of the Article.

