# Supplementary material for: High FMNL3 expression promotes nasopharyngeal carcinoma cell metastasis: role in TGF-β1-induced epithelia-to-mesenchymal transition
Source: Sci Rep. 2017 Feb 15;7:42507. doi: 10.1038/srep42507 (PMC5309845; doi:10.1038/srep42507)
Supplement: Supplementary Information [file srep42507-s1.pdf]

Supporting Information

High FMNL3 expression promotes nasopharyngeal carcinoma cell metastasis: role in TGF-β1-induced epithelia-to-mesenchymal transition

Yanxia Wu[1,*], Zhihua Shen[2,*], Keke Wang[1,*], Yanping Ha[1], Hong Lei[1], Yanan Jia[2], Ranran Ding[1,3], Dongmei Wu[1], Siyuan Gan[1], Rujia Li[1], Botao Luo[1], Hanguo Jiang[1], Wei Jie[1]

[1]Department of Pathology, Guangdong Medical University, Zhanjiang 524023, China.

[2]Department of Pathophysiology, Guangdong Medical University, Zhanjiang 524023, China.

[3]Department of Pathology, Union Hospital, Tongji Medical College, Huazhong University of Science and Technology, Wuhan 430030, China.

[*]These authors contributed equally to this work

Correspondence and requests for materials should be addressed to W.J. (wei.jie@gdmu.edu.cn) or H.J. (Jianghg12@163.com)

Supporting Figures

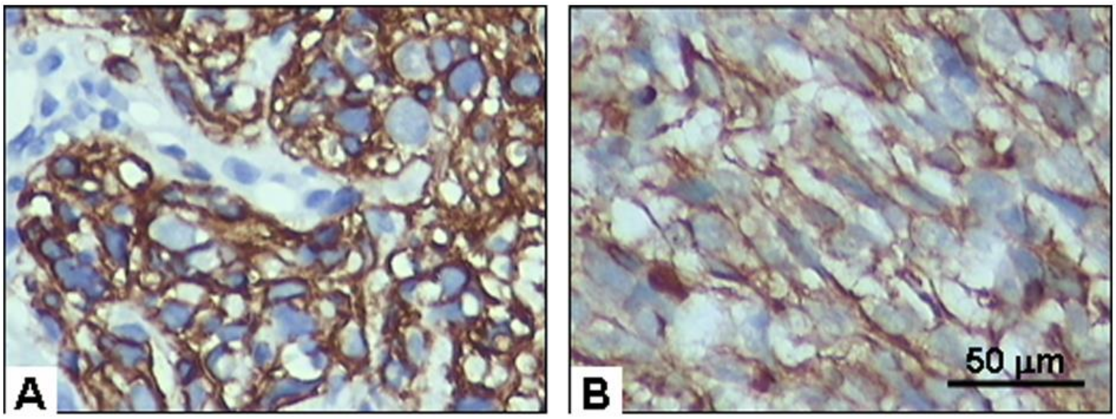

Figure S1. Representative images of cytosol E-cadherin and vimentin protein expression in clinical NPC tissues. The immunohistochemical PV9000 method was used to examine E-cadherin and Vimentin protein expression in clinical samples. **(A)** Cytosolic E-cadherin expression was found in tumor cells in NPC tissue. **(B)** Vimentin protein was expressed in spindle-like tumor cells in NPC samples. The expression antigens in cells was colorized with DAB and counterstained with hematoxylin. Original magnification, × 200. Scale bar, 50 µm.

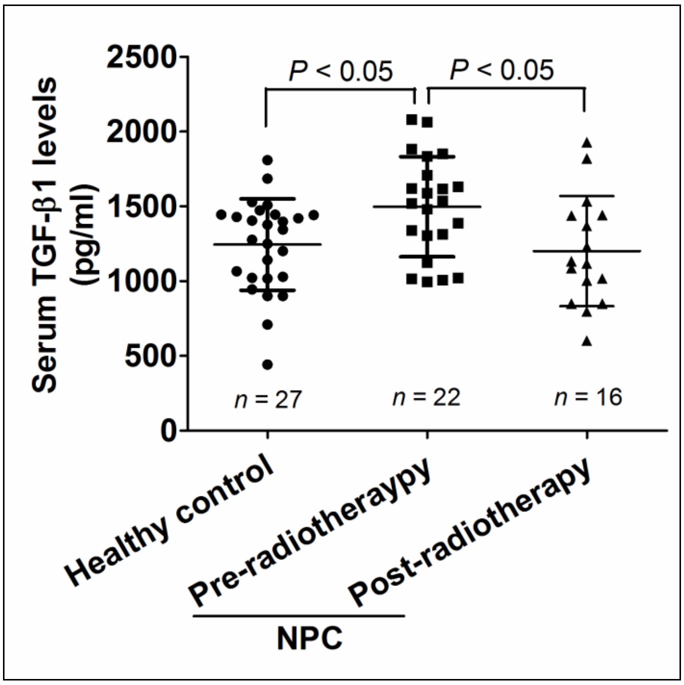

Figure S2. Serum TGF-β1 levels in samples of NPC patients. Serum samples were obtained from 38 NPC patients (27 men and 11 women; 22 pre-radiotherapy and 16 post-radiotherapy; median age of 48 years) and 27 normal controls (healthy donors; 14 men and 13 women; median age of 49 years) at the Affiliated Hospital of Guangdong Medical University during 2014. Enzyme-linked immunosorbent assay (ELISA) procedures to test serum TGF-β1 levels were performed according to the manufacturer's instructions (R&D System, MN, USA). The mean serum level of TGF-β1 in untreated NPCs was 1498±334.6 pg/ml, which was significantly higher than that in control samples (1245±305.6 pg/ml) ($p < 0.05$). However, the mean serum TGF-β1 level in NPC samples post-radiotherapy declined to 1201±368.4 pg/ml and was significantly lower than that of the pre-radiotherapy group ($p < 0.05$). Data were described as mean ± SD.

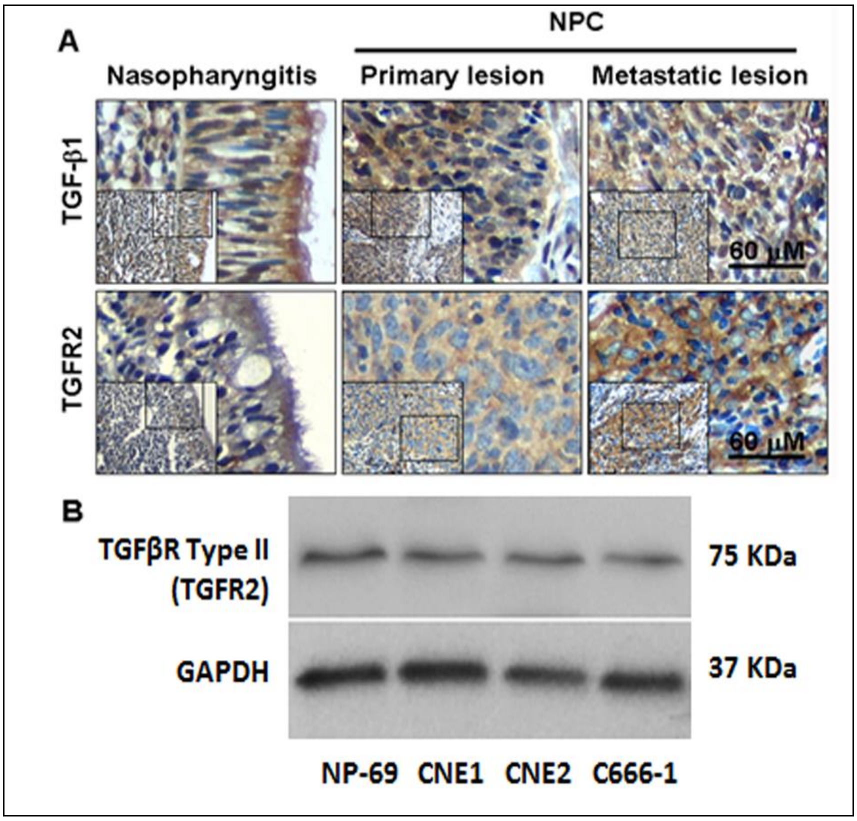

Figure S3. Expression of TGFβ1/TGFR2 in clinical samples and cell lines. (A) Representative images of down-regulation of TGF-β1 and up-regulation of TGFR2 (TGF-β receptor 2) protein in clinical nasoharyngitis and NPC tissues. The immunohistochemical PV9000 method was used to detect TGF-β1 and its receptor TGFR2 protein expression in clinical samples. Non-immune IgG was used as a negative control. The expression and location of TGF-β1 and TGFR2 in cells was colorized with DAB and counterstained with hematoxylin. Left panels, nasopharyngitis; middle panels, NPC primary lesions; right panels, NPC metastatic lesions. Original magnification, ×400. **(B)** Western blotting result of expression of TGFR2 protein in NP-69, CNE1, CNE2 and C666-1 cells.

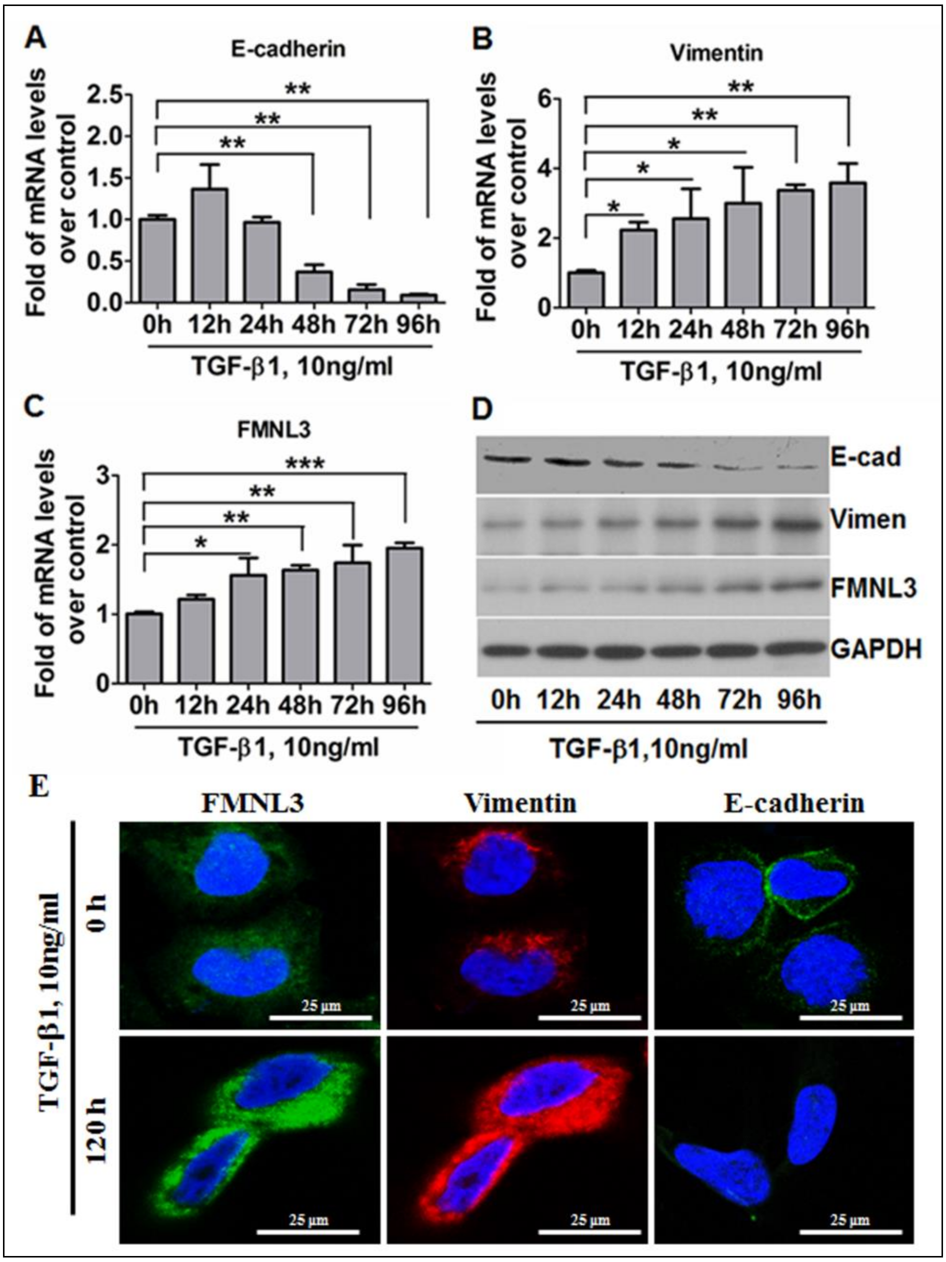

Figure S4. TGF-β1 induces the changes in the expression EMT markers and FMNL3 in CNE2 cells at time-consuming manner. CNE2 cells were treated with 10 ng/ml TGF-β1 for 0, 12, 24, 48, 72, 96 and 120 hours, cells were then harvested. The changes in E-cadherin (E-cad), Vimentin (Vimen) and FMNL3 were assessed by qRT-PCR **(A, B** & **C**) and western blotting **(D)**. GAPDH was used as intern control. **P*<0.05; ***P*<0.01; ****P*<0.001. **(E)** Immunofluorescence staining of FMNL3, Vimentin and E-cadherin in untreated or TGF-β1-treated CNE2 cells. Nuclei were counterstained with DAPI.

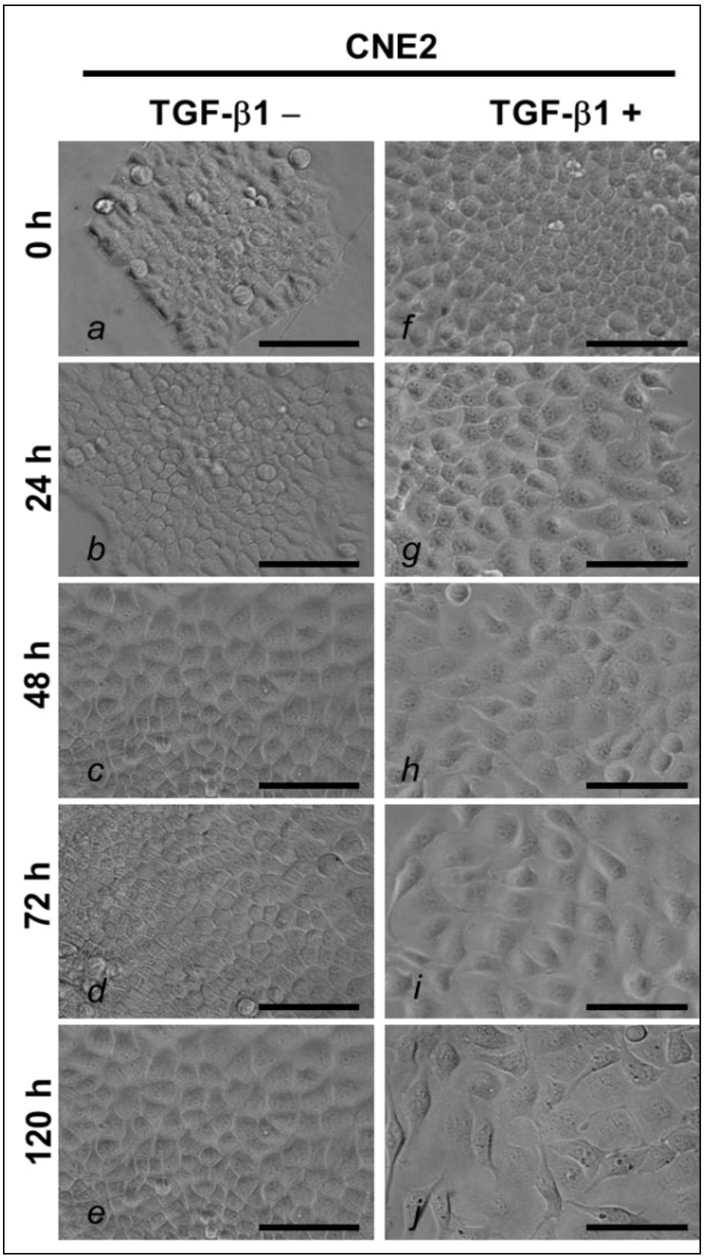

Figure S5. Representative images of the morphological changes of CNE2 cells exposed to exogenous TGF-β1. CNE2 cells were used to observe whether TGF-β1 treatment induced the morphological changes of EMT. After treating highly FMNL3-expressing CNE2 cells without (*a* – *e*) or with (*f* – *j*) exogenous TGF-β1 (10 ng/ml, Cat. #10021, PropTech, NJ, USA) for various periods, the cells were observed and photographed under a microscope. Scale bar, 60 μm.

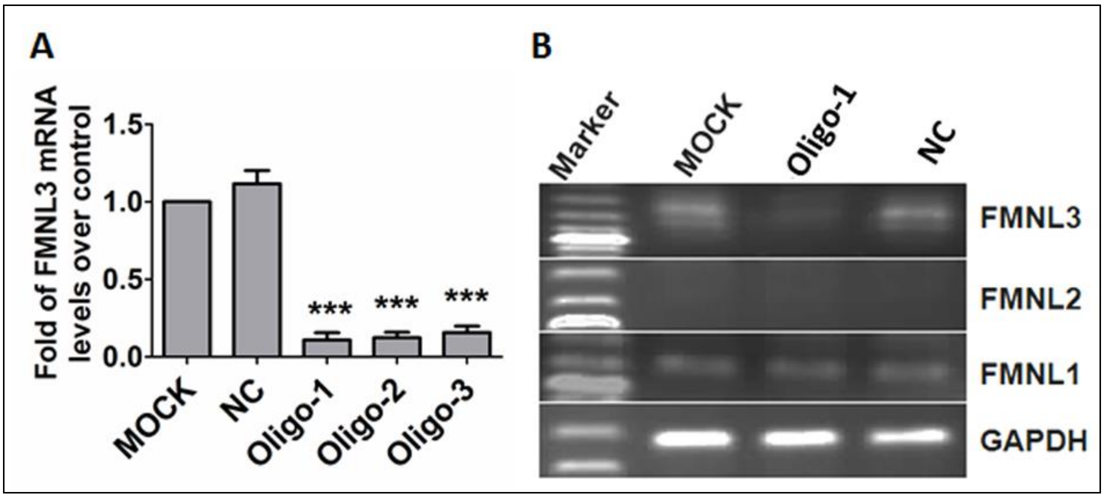

Figure S6. FMNL3 specific siRNA attenuates FMNL3 expression in CNE2 cells. (A) CNE2 cells were transduced with FMNL3 specific siRNAs (oligo-1, -2 and -3) for 48 h, and then cells were harvested for qRT-PCR analysis of the FMNL3 mRNA levels, cells without oligo transduction (MOCK) or cells transduced with negative control oligo (NC) was as controls. ****P*<0.001 vs. MOCK or NC. **(B)** CNE2 cells were transduced with Oligo-1 for 48h, semi-quantitative RT-PCR was used to test mRNA levels for FMNL3, FMNL2 and FMNL1. FMNL3 specific siRNA knocked down FMNL3 mRNA significantly; however it showed no effects on the change of FMNL1 mRNA. There was no detectable FMNL2 mRNA in CNE2 cells. Primers for FMNL3 (5'-3 '), cagcgaacttgatgatgagaag, tcttgtttttggagcagatgag; Primers for FMNL2 (5'-3'), gcaggtaactctgtgggacc, acagctgctaatcctgagttga; Primers for FMNL1 (5'-3'), tgtggatactggtggggtca, tggggtcagcttgatggtca; Primers for GAPDH (5'-3'), gtcaacggatttggtcgt, ttgattttggaggatctcg.

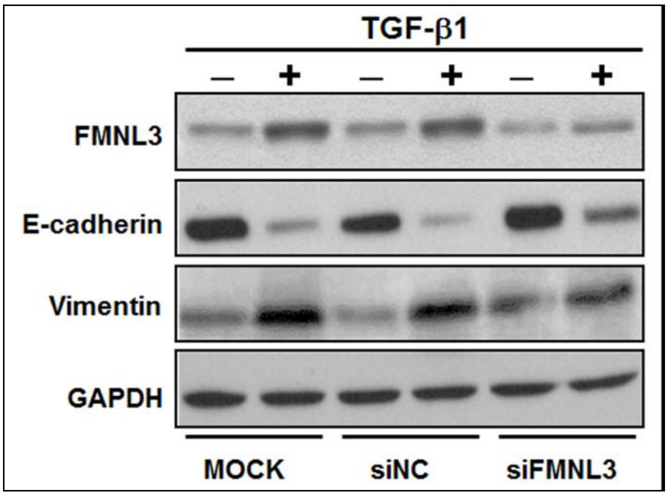

Figure S7. Knockdown of FMNL3 expression alters FMNL3 and EMT marker gene expression in CNE2 cells. CNE2 cells were transfected with FMNL3-specific siRNA (siFMNL3, oligo 1), control siRNA (siNC), or no siRNA (MOCK), and then treated with or without 10 ng/ml TGF-β1 for 48 h. The cells were harvested and analyzed to detect FMNL3, E-cadherin, and Vimentin expression by Western blot.

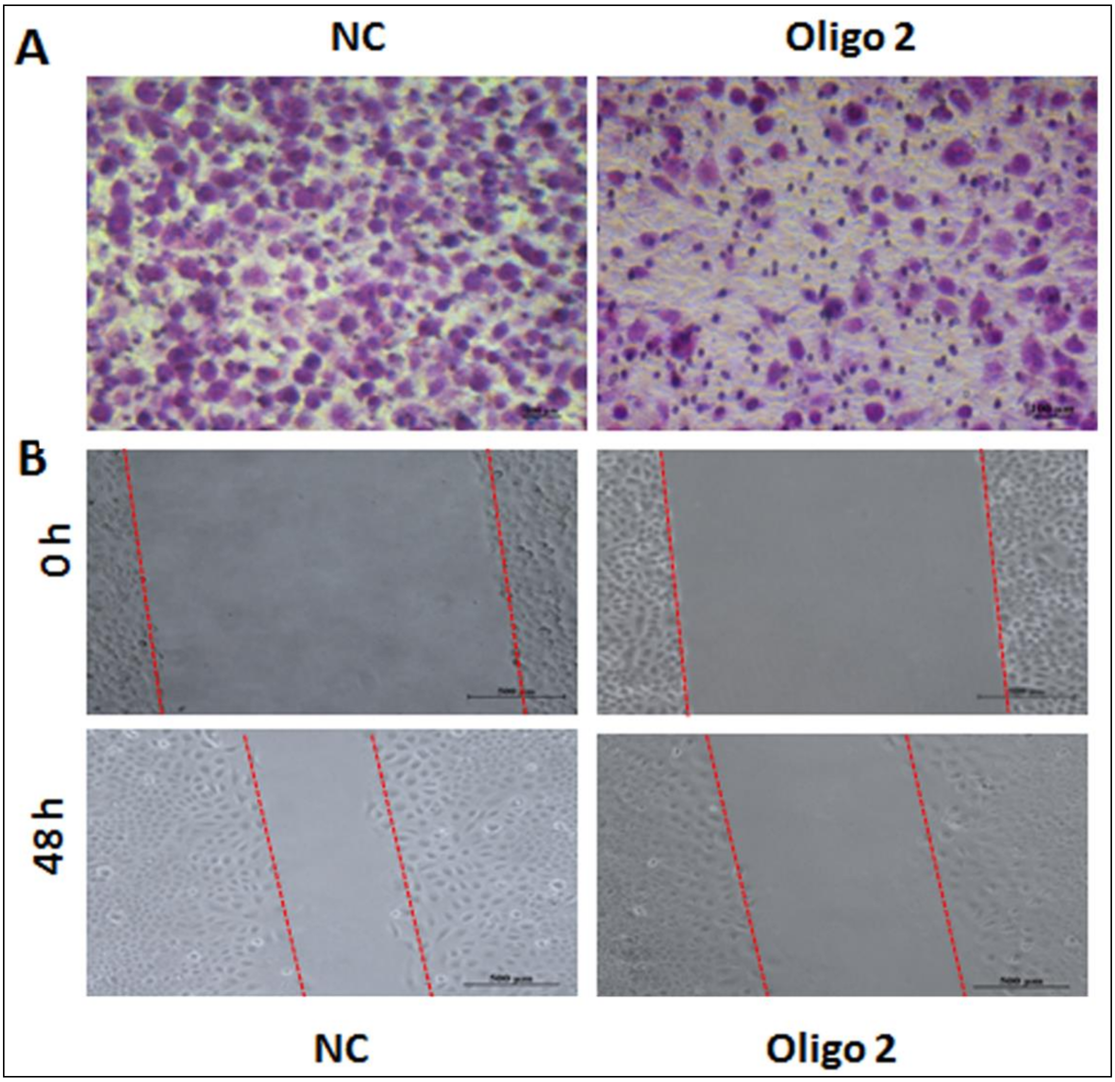

Figure S8. Knockdown of FMNL3 expression with oligo 2 suppresses CNE2 cells' ability in migration. CNE2 cells were transfected with FMNL3-specific siRNA (oligo 2) and control siRNA (NC) for 48 h without addition of TGF-β1 (10 ng/ml), then cells were subjected to **(A)** transwell assay and **(B)** wound healing assay. For transwell assay, 1×10^5 cells in 250 μl DMEM with 1% FBS were added in upper chamber, and 500 μl DMEM supplemented with 10% FBS was added to the lower chamber. Migration was allowed to proceed for 25 h at 37 ℃; membranes were fixed for 20 min with 4% neutral formalin and stained with 0.1% crystal violet for 10 min, and the migrated cells on the lower surfaces of the membranes were photographed. Each group in transwell assay and wound healing assay was repeated three wells. Original magnification for (A), ×200; for (B), ×100.

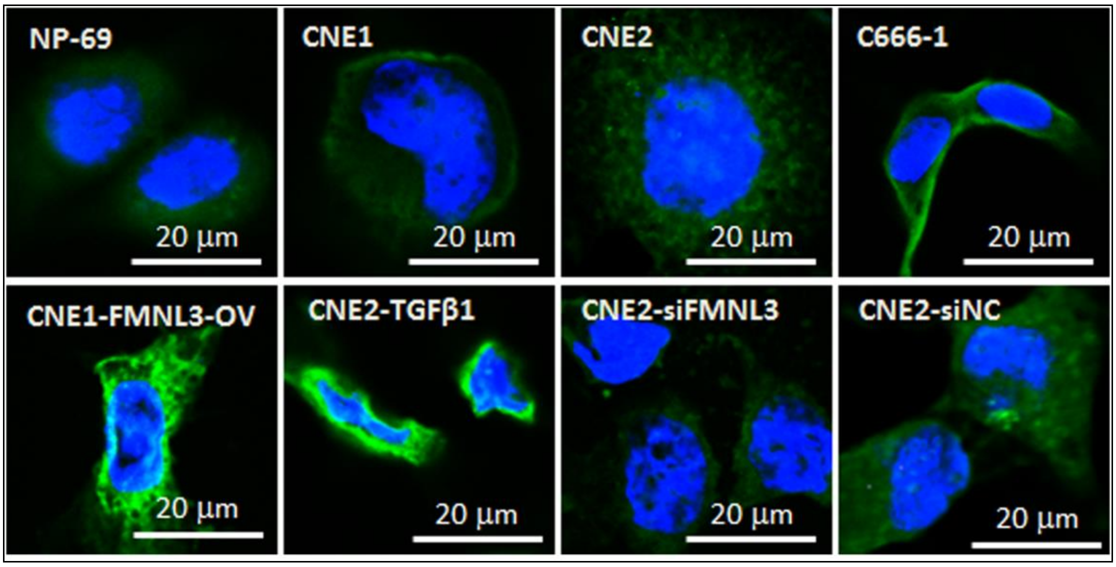

Figure S9. Filamentous actin (F-actin) staining in immortalized nasopharyngeal epithelial cell line NP69 and nasopharyngeal carcinoma cell lines. NP-69, CNE1, CNE2, C666-1, CNE1-FMNL3-OV (FMNL3-overexpressing CNE1 cells, 72 h), CNE2-TGF-β1 (TGF-β1-treated CNE2 cells, 10 ng/ml, 120 h), CNE2-siFMNL3 (siRNA-transfected CNE2 cells, 48 h) and CNE2-siNC (negative control siRNA-transfected CNE2 cells, 48 h) cells were subjected F-actin staining by immunofluorescence staining using FITC-conjugated Phalloidin (Sigma, #Cat. P5282). Nuclei were counterstained with DAPI.

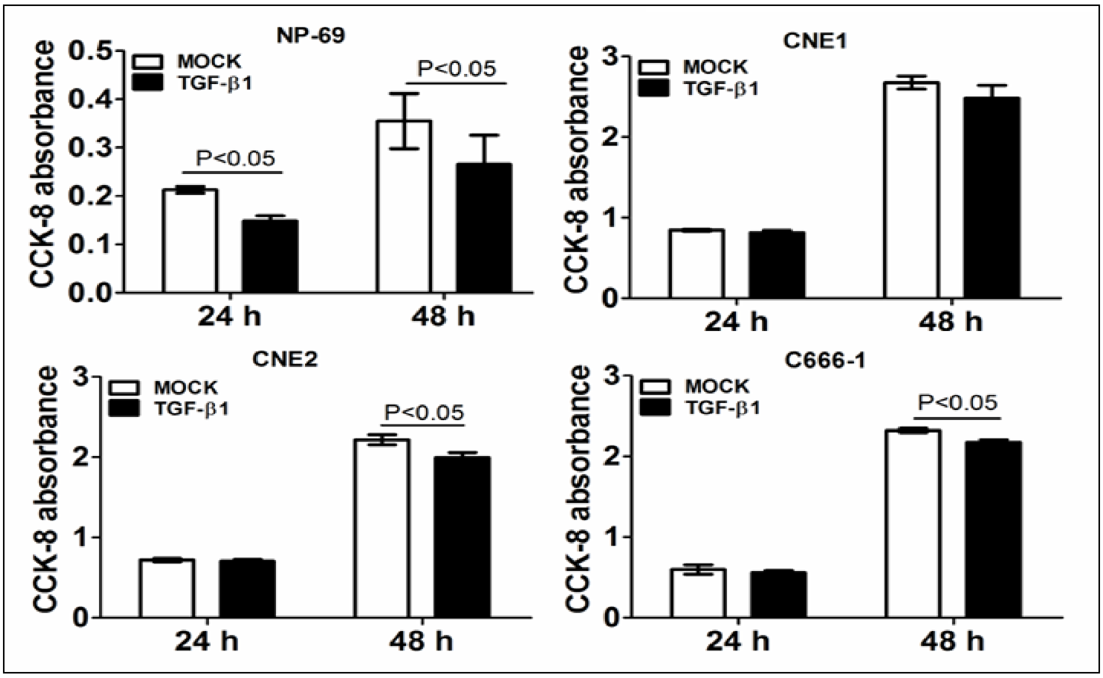

Figure S10. Exogenous TGF-β1 represses NPC cell proliferation in vitro. *In vitro* cell proliferation was assessed using the Cell Counting Kit-8 (CCK-8) assay. Briefly, NP-69, CNE1, CNE2, or C666-1 cells (2×10^3 per well) were seeded in 96-well plates overnight, and then the cells were treated with 10 ng/ml TGF-β1 (Cat. #10021, PropTech, NJ, USA) for 24 or 48 h. After washing with 1 ×PBS, 10 μl CCK-8 reagent (Biyuntian, Jiangsu, China) in 100 μl DMEM was added per well, and the cells were incubated at 37℃ for 2 h. Cell viability was represented by the optical density measured using a microplate reader. Each group was repeated 6 wells, and data represent the mean ±SD of three independent experiments.

Table S1. The correlations between FMNL3 and E-cadherin, FMNL3 and Vimentin in clinical NPC samples

FMNL3	E-cadherin				*r*	*P*	Vimentin				*r*	*P*
	-	+	++	+++			-	+	++	+++		
-	19	7	6	7	-0.292	0.001	23	13	3	0	0.236	0.010
+	14	3	1	1			2	12	4	1		
++	22	10	1	0			6	23	3	1		
+++	21	5	1	1			6	19	2	1		

The expressions of FMNL3, E-cadherin and Vimentin protein in NPC samples (n = 119) were detected by immunohistochemical staining. Non-immune IgG was used as a negative control. Antigenic sites were visualized using a PV9000/9003 and DAB kits. The immunoreactive scores (IRSs) of FMNL3, E-cadherin and Vimentin were calculated as follows: 0, negative; 1, weak; 2, moderate; 3, strong. The percentage of positive cells was scored as 1, 0–9% positive cells; 2, 10–50% positive cells, and 3, >50% positive cells. Samples with a total IRS of 0, 1-3, 4-6 and 7-9 were considered to be (–), (+), (++) and (+++) of FMNL3, E-cadherin or Vimentin. The correlation between FMNL3 and E-cadherin, FMNL3 and Vimentin was analyzed by Spearman correlation analysis method.

Table S2. The correlation of E-cadherin to Vimentin in clinical NPC samples

E-cadherin	Vimentin				*r*	*P*
	-	+	++	+++		
-	16	51	7	2	-0.207	0.024
+	11	9	4	1		
++	6	2	1	0		
+++	4	5	0	0		

The expressions of E-cadherin and vimentin protein in NPC samples (n = 119) were detected by immunohistochemical staining. Non-immune IgG was used as a negative control. Antigenic sites were visualized using a PV9000/9003 and DAB kits. The immunoreactive scores (IRSs) of E-cadherin and Vimentin were calculated as follows: 0, negative; 1, weak; 2, moderate; 3, strong. The percentage of positive cells was scored as 1, 0–9% positive cells; 2, 10–50% positive cells, and 3, >50% positive cells. Samples with a total IRS of 0, 1-3, 4-6 and 7-9 were considered to be (–), (+), (++) and (+++) of E-cadherin or Vimentin. The correlation between E-cadherin and Vimentin was analyzed by Spearman correlation analysis method.
